# Two types of human Th17 cells with pro- and anti-inflammatory properties and distinct roles in autoinflammation

**DOI:** 10.1186/1546-0096-13-S1-O49

**Published:** 2015-09-28

**Authors:** R Noster, H de Koning, F Sallusto, C Zielinski

**Affiliations:** 1 Institute for Medical Microbiology, Immunology and Hygiene, München, Germany; 2 Charité-Universitätsmedizin Berlin, Berlin, Germany; 3 Radboud University Nijmegen Medical Centre, Nijmegen, Netherlands; 4 Institute for Research in Biomedicine, Bellinzona, Switzerland

## Introduction

Th17 cells are known to be crucial mediators of autoimmune inflammation. However, two distinct types of Th17 cells have recently been described, which differed in their ability to coproduce IL-10 or IFN-g due to differential polarizations requirements for IL-1b. Whether these distinct Th17 phenotypes translate into distinct Th17 cell functions and whether this has implications for human health or disease has not been addressed yet.

## Objectives

We hypothesized that IL-1b independent IL-10^+^Th17 cells have anti-inflammatory functions whereas IL-1b dependent IL-10^-^ Th17 cells are pro-inflammatory. Considering the crucial role of IL-1b in the pathogenesis of autoinflammatory syndromes, we hypothesized an IL-1b mediated loss of anti-inflammatory Th17 cell functions in Schnitzler Syndrome, an autoinflammatory disease.

## Methods

To assess pro- versus anti-inflammatory Th17 cell functions we performed suppression assays and tested the effects of IL-1b dependent and independent Th17 subsets on modulating pro-inflammatory cytokine secretion by monocytes. Schnitzler Syndrome patients were analyzed for changes in Th17 cell functions before and after therapy with IL-1b depleting drugs.

## Results

IL-10^+^ Th17 cells, which differentiated independently of IL-1b, have regulatory functions similar to Treg cells while IL-1b dependent IL-10^-^ Th17 cells have not. Both Th17 cell subsets differ in their ability to suppress T cell proliferation as well as in their ability modulate pro-inflammatory cytokine production by antigen presenting cells. In Schnitzler Syndrome, an autoinflammatory syndrome, overproduction of IL-1b translates into pro-inflammatory Th17 cell functions, which can be reversed by anti-IL-1b treatment.

## Conclusion

Th17 cells are not *per se* pro-inflammatory but can also have anti-inflammatory IL-10 mediated functions if generated independently of IL-1b. Our data introduce Th17 cell subsets as novel players in autoinflammation and thus novel therapeutic targets in autoinflammatory syndromes including other IL-1b mediated diseases. This demonstrates for the first time alterations in the adaptive immune system in autoinflammatory syndromes.

